# Plasma Protein Levels Analysis in Multiple Sclerosis Sardinian Families Identified C9 and CYP24A1 as Candidate Biomarkers

**DOI:** 10.3390/life12020151

**Published:** 2022-01-20

**Authors:** Andrea Nova, Teresa Fazia, Ashley Beecham, Valeria Saddi, Marialuisa Piras, Jacob L. McCauley, Carlo Berzuini, Luisa Bernardinelli

**Affiliations:** 1 Department of Brain and Behavioral Sciences, University of Pavia, 27100 Pavia, Italy; teresa.fazia01@ateneopv.it (T.F.); luisa.bernardinelli@unipv.it (L.B.); 2 John P. Hussman Institute for Human Genomics, Miller School of Medicine, University of Miami, Miami, FL 33146, USA; ABeecham@med.miami.edu (A.B.); jmccauley@med.miami.edu (J.L.M.); 3 Dr. John T. Macdonald Foundation Department of Human Genetics, Miller School of Medicine, Miami, FL 33136, USA; 4 Divisione di Neurologia, Presidio Ospedaliero S. Francesco, ASL Numero 3 Nuoro, 08100 Nuoro, Italy; valeria.saddi@tiscali.it (V.S.); marialpiras@tiscali.it (M.P.); 5 Centre for Biostatistics, The University of Manchester, Manchester M13 9PL, UK; carlo.berzuini@manchester.ac.uk

**Keywords:** biomarkers, plasma proteins, family data, suspension bead array technology, explained variability

## Abstract

Here we investigate protein levels in 69 multiple sclerosis (MS) cases and 143 healthy controls (HC) from twenty Sardinian families to search for promising biomarkers in plasma. Using antibody suspension bead array technology, the plasma levels of 56 MS-related proteins were obtained. Differences between MS cases and HC were estimated using Linear Mixed Models or Linear Quantile Mixed Models. The proportion of proteins level variability, explained by a set of 119 MS-risk SNPs as to the literature, was also quantified. Higher plasma C9 and CYP24A1 levels were found in MS cases compared to HC (*p* < 0.05 after Holm multiple testing correction), with protein level differences estimated as, respectively, 0.53 (95% CI: 0.25, 0.81) and 0.42 (95% CI: 0.19, 0.65) times plasma level standard deviation measured in HC. Furthermore, C9 resulted in both statistically significantly higher relapsing-remitting MS (RRMS) and secondary-progressive MS (SPMS) compared to HC, with SPMS showing the highest differences. Instead, CYP24A1 was statistically significantly higher only in RRMS as compared to HC. Respectively, 26% (95% CI: 10%, 44%) and 16% (95% CI: 9%, 39%) of CYP24A1 and C9 plasma level variability was explained by known MS-risk SNPs. Our results highlight C9 and CYP24A1 as potential biomarkers in plasma for MS and allow us to gain insight into molecular disease mechanisms.

## 1. Introduction

Multiple sclerosis (MS) (OMIM 126200) is an inflammatory demyelinating disorder of the central nervous system (CNS) and the most frequent cause of chronic neurological disability [1,2,3]. Epidemiological studies have provided evidence on the multifactorial nature of MS susceptibility involving both genetic and environmental factors [4,5]. In the disease process several pathophysiological mechanisms, including inflammation, demyelination, axonal damage, and repair mechanisms are involved. These mechanisms are not uniformly represented across patient populations, thus contributing to the heterogeneity in phenotypic expression of the disease, its prognosis, and response to therapies [6]. Thus, the need for suitable biomarkers to assist in the treatment decisions and in the choice of effective therapy strategy is compelling.

As defined in [7], “[a] biomarker is a characteristic that is objectively measured and evaluated as an indicator of normal biological process, pathogenic processes, or pharmacologic responses to a therapeutic intervention”.

For neurological diseases such MS, cerebrospinal fluid (CSF), given its proximity to the CNS, would be the preferred body fluid in which to search for candidate biomarkers rather than plasma or serum. Clearly, however, the CSF sample collection is a more invasive procedure with potential risks compared to plasma sampling. For this reason, CSF samples from healthy individuals are seldom taken especially for discovery-driven approaches. On the other hand, the presence in blood plasma of leakage or secretion products from various tissues or blood cells can reflect the pathological and physiological state of certain tissues [8] so that plasma, easily obtained through a non-invasive procedure, can be considered a good body fluid on which to search for promising diagnostic biomarkers.

Proteome in plasma has started to be studied in a high throughput manner, thanks to the technological development of the Human Protein Atlas (HPA) project (https://www.proteinatlas.org/, accessed on 12 July 2021) which has allowed the production of a large resource of polyclonal antibodies [9].

In the past years, taking advantage of new proteomic technologies [10], the field of biomarker discovery has gradually shifted from the aim of finding a unique perfect surrogate marker to the construction of a composite panel of candidate biomarkers with higher performance, particularly suitable in the context of complex multifactorial diseases, such as MS, where many factors are involved in the development and progression of the disease.

Despite many efforts, the identification of a suitable set of biomarkers for MS based on peripheral blood is just beginning due to the complexity of MS [11]. Affinity proteomic methods have been used to identify MS-related proteins both in plasma and in CSF, leading to the identification of IRF8, IL7, SLC30A7, METTL14, and GAP43 [12,13,14,15,16,17,18]. A recent work [19], using antibody suspension bead array, has led to the identification of nine proteins to be regarded as potentially useful biomarkers for MS treatment, where plasma levels decrease after treatment with natalizumab, with two of these, PEBP1 and RTN3, showing the most significant changes.

However, very few of the many proposed candidate molecular MS biomarkers, specifically antibodies measuring humoral immune response (i.e., anti-natalizumab antibodies, neutralizing antibodies to interferon beta, IgG, anti-aquaporin-4 antibodies, and antibodies against varicella zoster and John Cunningham viruses) [20,21], have been validated and used in clinical practice, thus affecting clinical decision making [22].

Our study is framed within the field of biomarker discovery. More specifically, it was focused on the identification of new putative candidate biomarkers (proteins) for MS in plasma with the aim of gaining more insight into the pathogenic aspect of MS. To this aim we used protein profile data obtained via antibody suspension bead affinity-based array technology [23] from MS subjects and healthy related controls belonging to twenty multiplex families from the founder population of the Nuoro province, Sardinia (Italy).

The Mediterranean island of Sardinia is characterized by an age- and sex-adjusted prevalence of MS of 330 per 100,000 inhabitants, among the highest reported worldwide [24]. Furthermore, being a founder population isolated in a geographical, cultural, or religious sense, evolved from a small number of ancestors, subjected to limited immigration and hence characterized by high genetic homogeneity, it is the ideal population for the identification of disease determinants [25,26].

Our study was mainly structured into two phases. In the first phase we compared, in affected and non-affected individuals, 56 plasma protein profiles, a priori selected for their suggested association with MS from previous studies, to explore the possibility of identifying a set of reliable disease-specific diagnostic biomarkers. The highlighted proteins were further investigated by evaluating protein level differences between the MS disease-course classes.

In the second phase, we selected a panel of MS-risk genetic variants highlighted by the last study of the International Multiple Sclerosis Genetic Consortium (IMSGC) [27] to quantify the plasma level variability explained by these genetic variants in a colocalization perspective. If a consistent portion of variability can be explained by these MS-risk variants, this would add more evidence to the validity of our discovered biomarkers, and at the same time a potential biological function could be attributed to these variants. The rationale behind this second phase was that signals highlighted by Genome-Wide Association Studies (GWAS) are enriched with variants that could influence the levels of quantitative traits acting as quantitative trait loci (QTL), often in a tissue-specific manner. In fact, over three quarters of GWAS hits appear not to be associated with potentially deleterious protein-coding variants [28,29,30]. Therefore, the identified MS-risk variants can act as regulators of plasma protein levels and altogether explain proteins’ variability.

In summary, our analysis allows us to gain more insights into the understanding of molecular mechanisms underlying MS, to identify potential biomarkers for the disease, and to quantify proteins’ plasma level variability as explained by a set of well-established MS-risk SNPs to gain insight on their biological function and molecular disease mechanisms.

## 2. Results

### 2.1. Sample Description

Twenty Sardinian pedigrees, containing from 6 to 66 subjects (median = 22 subjects) and from 2 to 15 MS patients (median = 3 cases), were selected for the analysis, for a total of 533 subjects (96 affected and 437 healthy controls). For this study, families were further divided into 69 sub-families containing from 3 to 25 subjects (median = 7), to better capture the shared environment effect. Moreover, 56 antibodies targeting unique plasma protein levels (listed in Table S1), made available from the HPA project, and 131.497 genotyped variants from ImmunoChip, were available as follows:

- 212 subjects (58 sub-families, 69 MS cases, 143 healthy controls) had protein levels measured.
- 272 subjects (53 sub-families, 58 MS cases, 214 healthy controls) had ImmunoChip data.
- 135 subjects (43 sub-families, 43 MS cases, 92 healthy controls) had both protein levels and ImmunoChip data
- 77 subjects (29 sub-families, 26 MS cases, 51 healthy controls) had protein levels only
- 137 subjects (47 sub-families, 15 MS cases, 122 healthy controls) had ImmunoChip data only.
- 184 subjects (62 sub-families, 12 MS cases, 172 healthy controls) had neither protein levels nor ImmunoChip data.

Table 1 reports descriptive statistics for sex, age at the day of blood sampling, age at MS onset, and MS disease course for the 212 subjects having protein levels.

### 2.2. Differences in Protein Levels

#### 2.2.1. Power Evaluation

The primary endpoint was to detect significant protein level differences between MS cases and healthy controls. The statistical power to detect effect sizes ≥0.3 and ≥0.5 (in terms of healthy controls standard deviation (SD) scale) was evaluated using a simulation approach for both the linear mixed model (LMM) and linear quantile mixed model (LQMM) (this latter to be used in case of non-normality of LMM residuals). An effect size of 0.5 was chosen in the spirit of the distributional methods to define the minimum clinical important difference (MCID) as 0.5 times the SD of the endpoint of interest [31], even if in this case it is simply a measure of a potentially meaningful physiological change. The sample size was fixed to 212, the proportion of MS cases to 0.32 (as in our sample), and the pedigree structure as that of our Sardinian families. In the end 206 subjects were analyzed, since 6 subjects had a missing age at blood sampling day. Simulating protein levels 2000 times, the power to detect an effect size ≥0.5 at significance level α = 0.05 was >90% for both LMM and LQMM, while the power to detect an effect size ≥0.3 at α = 0.05 was, still, >90% for LMM but ≈60% for LQMM.

#### 2.2.2. Protein Levels Significantly Different between MS Cases and Healthy Controls

Differences between MS cases and healthy controls were evaluated for the 56 protein levels in 69 MS cases and 143 unaffected controls, belonging to 58 Sardinian sub-families, by fitting LMM as in Equation (1). Six subjects with missing age at blood sampling were excluded from the analysis. The normality of residuals was checked following Kim guidelines [32] and only in 14 models was the normality assumption respected. Thus, for the remaining 42 protein levels, LQMM was performed. Statistically significant results, after family-wise error (FWER) = 0.05 control using Holm procedure, obtained both fitting LMM or LQMM are reported in Table 2. Estimates represent the protein level difference between MS cases and healthy controls, adjusted for sex, age at blood sampling, kinship effect and shared environment effect, expressed as the number of SDs of the protein levels in healthy controls; thus, all estimates are to be considered on the healthy control SD scale (HC SD). As shown in Table 2, two proteins, complement C9 and cytochrome P450 family 24 subfamily A member 1 (CYP24A1), were significantly different between MS cases and healthy controls, after multiple testing Holm correction, and thus they were considered as potential biomarkers. Among the results not statistically significant, after multiple testing correction, it is worth mentioning three additional proteins considered suggestive, i.e., somatostatin (SST), ceruloplasmin (CP), and tissue type plasminogen activator (PLAT), having a sex- and age-adjusted absolute difference between MS case and healthy control levels of at least 0.30 HC SD with an uncorrected *p*-value < 0.005. Complete results for all 56 protein levels are provided in Supplementary Table S2.

**Figure 1:**
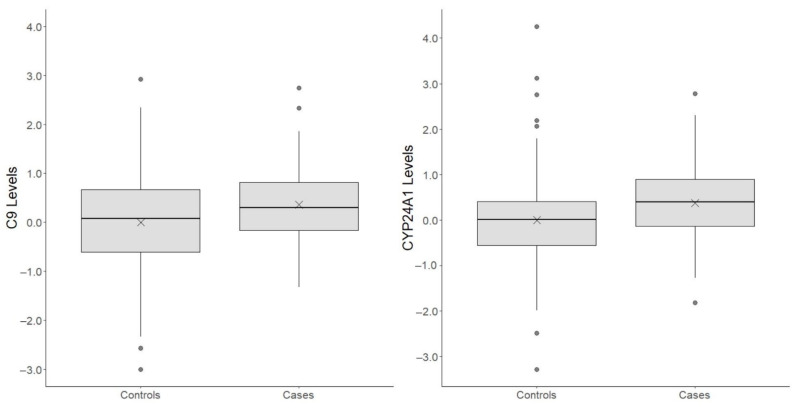
Box plots for statistically significant protein level differences between MS cases and healthy controls. Plasma protein levels were standardized using mean and standard deviation from healthy control protein levels; thus, 1 unit on the y-scale represents 1 standard deviation in healthy control protein levels. “X” represents the mean protein level within a group.

Specifically, C9 protein levels in MS cases were estimated, using LMM, on average 0.53 HC SD higher as compared to healthy control (95% CI: 0.25, 0.81) with a corrected *p*-value < 0.012. A CYP24A1 estimate was obtained through LQMM and, thus, is referred to the median (50th quantile) rather than the mean of protein levels. Therefore, the median of CYP24A1 protein levels in MS cases was 0.42 HC SD higher compared to healthy controls (95% CI: 0.19, 0.65) with a corrected *p*-value < 0.023. Box plots, for these two plasma protein levels, are provided in Figure 1.

**Figure 2:**
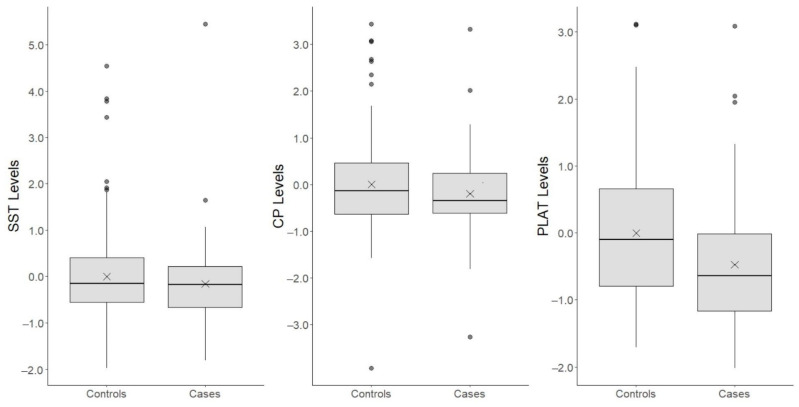
Box plots for SST, CP, and PLAT protein level differences between MS cases and healthy controls. Plasma protein levels were standardized using mean and standard deviation from healthy control protein levels; thus, 1 unit on the y-scale represents 1 standard deviation in healthy controls’ protein levels. “X” represents the mean protein level within a group.

**Figure 3:**
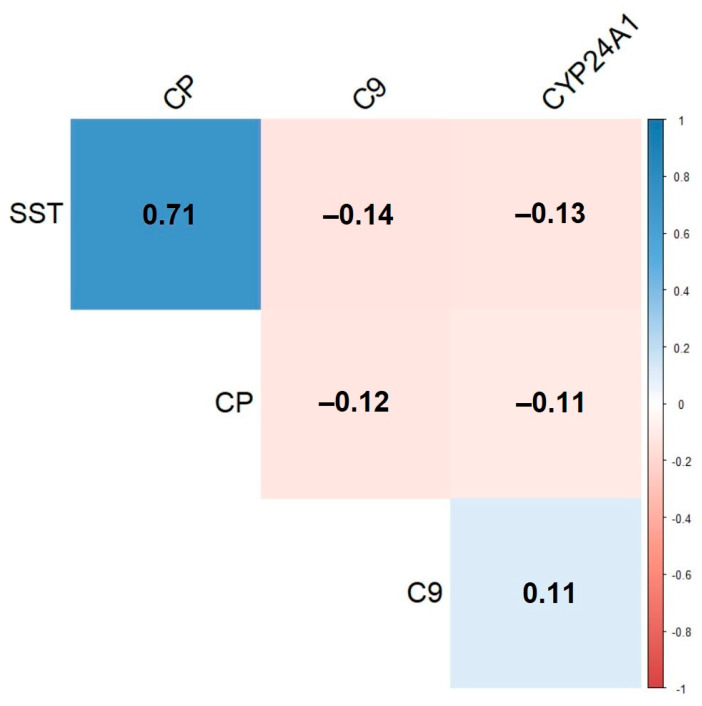
Pearson pairwise correlations between C9, CYP24A1, SST, CP, and PLAT plasma protein levels.

The sex- and age-adjusted estimated median of SST and CP plasma protein levels was lower in MS cases, i.e., −0.37 HC SD (95% CI: −0.62, −0.12, uncorrected *p* < 0.004) and −0.38 HC SD (95% CI: −0.64, −0.13, uncorrected *p* < 0.004), respectively. Additionally, PLAT plasma protein levels were lower, on average, in MS cases, i.e., −0.32 HC SD (95% CI: −0.54, −0.10, uncorrected *p* < 0.004). Box plots, for these three plasma protein levels, are provided in Figure 2. Pearson’s coefficient correlation matrix for C9, CYP24A1, SST, CP, and PLAT plasma protein levels is depicted in Figure 3. SST and CP were highly correlated, having Pearson’s coefficient *r* = 0.71, while the other proteins were weakly correlated, i.e., *r* < 0.15.

CYP24A1, SST, and CP protein level differences between MS cases and healthy controls have also been explored at the 25th and 75th quantiles of the protein level distribution (Table 3). The results obtained at these quantiles were almost identical to those of the 50th quantile, meaning that the non-normality of residuals obtained using linear regression to infer plasma protein levels’ entire distribution did not meaningfully impact estimates at different quantiles. Therefore, CYP24A1, SST, and CP protein level differences between MS cases and healthy controls at the median could be generalized to higher and lower levels of the distribution.

The five proteins were further explored evaluating protein level differences (sex- and age-adjusted) between the MS course classifications. Relapse-remitting MS (RRMS) and secondary progressive MS (SPMS) were compared to healthy controls (HC), and RRMS and SPMS cases were compared to each other. Results are reported in Table 4 and the number of subjects included in these analyses are summarized as follows (omitting subjects with missing age at blood sampling day):

- RRMS vs. HC comparisons comprised 181 subjects (41 RRMS cases and 140 healthy controls)
- SPMS vs. HC comparisons comprised 158 subjects (18 SPMS cases and 140 healthy controls)
- SPMS vs. RRMS comparisons comprised 59 subjects (18 SPMS cases and 41 RRMS cases)

C9 protein levels were significantly higher in both RRMS and SPMS cases compared to healthy controls (*p* < 0.05). On average, plasma C9 levels were, respectively, 0.44 HC SD and 0.62 HC SD higher in RRMS cases and SPMS cases compared to healthy controls. Both estimates were close to the estimate obtained considering overall MS cases (0.53 HC SD, see Table 3), with SPMS cases showing the highest differences.

**Figure 4:**
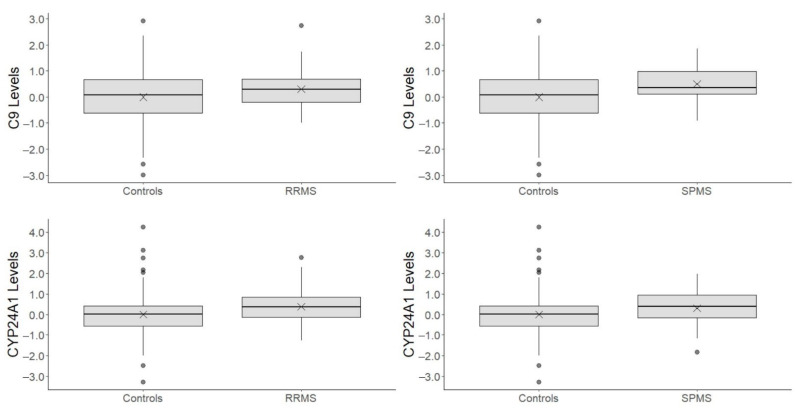
Box plots for C9 and CYP24A1 plasma protein level differences between relapse-remitting MS (RRMS) and secondary progressive MS (SPSM) compared to healthy controls. Plasma protein levels were standardized using mean and standard deviation from healthy controls’ protein levels; thus, 1 unit on the y-scale represents 1 standard deviation in healthy controls’ protein levels. “X” represents the mean protein level within a group.

Estimated median CYP24A1 protein levels resulted 0.46 HC SD higher (*p* < 0.05) in RRMS cases compared to healthy controls, while in SPSM cases the difference was lower, i.e., 0.32 HC SD (*p* > 0.05). As for C9, estimates were close to the estimate obtained considering overall MS cases (0.42 HC SD, see Table 3), with RRMS cases showing the highest difference. Boxplots are reported in Figure 4 for RRMS vs. HC and SPMS vs. HC comparisons in C9 and CYP24A1 plasma proteins. Regarding suggestive proteins SST and CP protein levels, SPMS cases’ protein levels in comparison with healthy controls showed a higher difference (in the median) compared to RRMS cases vs. healthy control comparison (−0.51 vs. −0.28 HC SD for SST, −0.40 vs. −0.28 HC SD for CP), with the former being statistically significant (*p* < 0.05). On the contrary, plasma PLAT levels’ median was significantly lower in RRMS cases compared to healthy controls (−0.44 HC SD, *p* < 0.05), with SMPS vs. HC PLAT difference being slightly lower in magnitude (−0.35, *p* > 0.05). Due to small sample size, i.e., *n* = 56, all comparisons between RRMS cases and SPMS cases resulted in estimated protein level differences being close to zero and in estimated large standard errors, making these comparisons not interpretable.

### 2.3. Results of Plasma Protein Levels Variability Explained by MS-Risk Variants

A total of 92 unaffected Sardinian controls (48 female and 44 males) from 39 sub-families were included in the subsequent analysis but one subject was omitted due to missing age at the day of blood sampling. The aim of this second analysis was to quantify the variability in protein levels in plasma, explained by well-known MS-risk genetic variants.

To this aim we firstly identified among a set of 139 MS-risk SNPs, as from the IMSGC study [27], those associated with the plasma levels of the two highlighted proteins (i.e., C9 and CYP24A1) in our sample. Of these 139 MS genetic risk variants, 119 were included in the analysis, 4 SNPs were removed due to linkage disequilibrium (*r*^2^ > 0.2) and 16 SNPs were removed due to minor allele frequency (MAF), <0.10 in our sample of 91 healthy controls. Firstly, 119 univariate models as in Equation (3) were performed for C9 and CYP24A1, for a total of 238 statistical tests. Secondly, among the statistically significant SNP–protein associations at α = 0.10, the best set of SNPs were selected using a stepwise regression procedure. Subsequently, a multivariable LMM model, which includes the best set of SNPs and which adjusts for age at blood sampling day, sex, kinship effect, and shared environment effect, was fitted to estimate the marginal proportion of protein level variability jointly explained by significant SNPs at α = 0.01 (*R*^2^_SNPs_); a 95% confidence interval was estimated as (bias-corrected accelerated) BCa interval, at α = 0.05, simulating 1000 bootstrap replications as explained in the Materials and Methods section. In Table 5 the results for *R*^2^_SNPs_, along with a 95% confidence interval and significant SNPs at α = 0.01, are reported. A total of 16% (95% CI: 9%, 39%) of plasma C9 level variability was jointly explained by two MS-risk SNPs, i.e., rs2104286 and rs1610555; their effect sizes have been estimated, respectively, as 0.61 (95% CI: 0.32, 0.90) and 0.32 (95% CI: 0.08, 0.56) HC SD, meaning that a single effect allele (the minor allele) was associated with an average increase of 0.61/0.32 HC SD, controlling for sex, age, kinship effect, shared environment effect, and the other SNPs included in the multivariable model. Since the model is additive and linear, a recessive homozygote genotype would then lead to doubled increases in C9 plasma levels, i.e., 1.22 and 0.64 HC SD.

As to the plasma CYP24A1 level, 26% (95% CI: 10%, 44%) of its variability was jointly explained by three MS-risk SNPs, i.e., rs1870071, rs11567694, and rs7665090, with the first explaining 12% of the total variability; their estimated effect sizes were, respectively, 0.64 (95% CI: 0.33, 0.96), 0.55 (95% CI: 0.23, 0.88) and −0.35 (95% CI: −0.60, −0.11) HC SD. Thus, a portion of both C9 and CYP24A1 plasma level variability was significantly explained by MS-risk SNPs. 

We have also explored *R*^2^_SNPs_ in the suggestive, but not significant after multiple testing correction, proteins found in the first analysis (see Section 2.2.2.), i.e., SST, CP, and PLAT (see Table 5). Plasma SST levels were not associated significantly with any MS-risk SNPs in the multivariable model. As to CP plasma levels, 16% (95% CI: 6%, 33%) of their variability was explained by three MS-risk SNPs, each of them explaining ≈ 5% of the total variability. Interestingly, for PLAT plasma levels, *R*^2^_SNPs_ were the highest among the five proteins analyzed, with almost one-third (32%, 95% CI: 13%, 49%) of plasma level variability explained by five significant MS-risk SNPs, among which rs11567694 explained the largest part (12%), with its effect allele resulting in an estimated PLAT level increase of 0.63 HC SD (95% CI: 0.24, 0.91). For all the five proteins, the 119 univariate and respective multivariate SNP–protein level associations are reported in Supplementary Table S3.

## 3. Discussion

MS is a heterogeneous disease, therefore reliable biomarkers that can capture the different aspects of this heterogeneity are required to understand its mechanism and pathophysiology, especially to diagnose, predict prognosis and response to therapies, and not least to develop new therapeutic strategies [22].

Our study tried to contribute to MS biomarkers research, specifically in blood plasma, where a set of well-established biomarkers has yet to be found and the research is still ongoing. In this work we have highlighted two proteins in plasma, i.e., C9 and CYP24A1, that could be regarded as putative diagnostic biomarkers for MS as their level in plasma was increased in affected subjects as compared to controls, which may serve as a good basis to extend the current understanding of the disease and the literature regarding biomarkers for MS in plasma. These findings could help to establish a set of biomarkers which could jointly discriminate between MS-affected and unaffected subjects with considerable accuracy [12]. C9 and CYP24A1 plasma levels were approximately 0.5 HC SD higher compared to healthy controls, therefore showing a clinically meaningful but “moderate” change [31], in line with results from previous studies searching for MS biomarkers from plasma protein levels [12,13,14,15,16,17,18]. 

Particularly, C9 plasma protein levels in MS cases were 0.53 HC SD higher (95% CI: 0.25, 0.81) compared to healthy controls, with a slightly higher difference found in SPMS cases compared with healthy controls (i.e., 0.62 HC SD, 95% CI: 0.18, 1.07). C9 is a constituent of the membrane attack complex (MAC); it plays a key role in the innate and adaptive immune response promoting phagocytic/endocytic elimination of non-self-antigens and immune complexes by inducing apoptosis [33]. In demyelinating disorders, such as MS, MAC could play a dual role, neurodegenerative or neuroprotective. Specifically, complement components, which constitute the MAC, can originate in plasma after the disruption of the blood brain barrier (BBB) or they can be synthesized locally in CNS cells, where they promote neuroinflammation and demyelination during the acute phase; on the other hand, during the chronic phase of the disease, they provide neuroprotection by promoting axon preservation, oligodendrocyte survival, and remyelination [34]. Therefore, the biological background supports our finding of increased C9 plasma levels in MS cases due to a systemic production of complement components consequent to a neuroinflammation state. Previous studies have investigated C9 level differences between MS cases and healthy controls in both CSF and plasma. 

While decreased C9 levels in CSF were found in different studies [35,36,37], Ingram et al. [35] was the only study to evaluate C9 plasma levels with a consistent sample size. The study [35] focused on the analysis of plasma complement components’ levels differences between MS cases and healthy controls, driven by a previous investigation which highlighted increased levels of complement components in MS cases’ plasma blood [38,39,40]. Their results showed a significant decrease for C9 plasma levels in both RRMS, SPMS cases, and overall MS cases, but at the same time significantly increased plasma levels of all the other complement proteins analyzed, i.e., C3 (where a difference between MS cases and healthy controls did not result significantly in our sample), C4, C4a, C1 inhibitor, and factor H were highlighted. Therefore, given that complement protein levels have been suggested as potential plasma biomarkers due to their increase in MS cases [35,38,39,40], a hint that is also supported by the biological background as described before [34], the finding in [35] does not necessarily contradict our result, but leads to the need to pinpoint plasma complement components levels, and therefore also plasma C9 levels, as candidate plasma MS biomarkers which should be further investigated in confirmatory studies. CYP24A1 plasma levels were higher, 0.42 HC SD (95% CI: 0.19, 0.65), as compared to healthy controls, with similar differences found in RRMS cases and SPMS cases comparisons with healthy controls. This mitochondrial protein initiates the degradation of calcitriol, the physiologically active form of vitamin D3, by hydroxylation of the side chain. In regulating the level of vitamin D3, this enzyme has a vital role in vitamin D metabolism [41]. Vitamin D deficiency has been highlighted as a risk factor for developing MS, and it was proposed primarily to explain the observation of a latitude gradient in MS prevalence. To support vitamin D deficiency as an MS risk-factor, MS-risk genetic polymorphisms have been discovered near to genes encoding key enzymes in vitamin D metabolism, and a low vitamin D status have been associated with a high risk of developing MS, high risk of MS relapses, and high level of MS-related disability [42]. Ramasamy et al. [43] found MS-risk SNP rs2248359, listed among the 200 prioritized MS-risk SNPs in the IMSGC meta-analysis study, to be strongly associated with increased gene expression of CYP24A1 in the frontal cortex, while two additional studies linked MS-risk SNPs rs2248137, located in CYP24A1 gene, to decreased serum vitamin D levels [41,44]. These findings led to the speculation that MS-risk genetic variants in the CYP24A1 gene could result in an increased expression or activity of this enzyme, leading to low circulating vitamin D levels. This claim is consistent with our finding of higher plasma CYP24A1 levels in MS cases compared to healthy controls, which could represent a risk factor, due to genetics or other unknown causes.

SST, CP, and PLAT have been selected among the suggestive, but not statistically significant after multiple correction, plasma protein levels different in MS cases compared to healthy controls. Therefore, it is important to underline that these discoveries are not controlled at fixed I type error (0.05) after multiple testing correction but could still give meaningful validations, about previous findings, and new unexplored insights. 

SST plasma levels were lower, 0.37 HC SD (95% CI: −0.62, −0.12), in MS cases compared to healthy controls, with a higher decrease found in SPMS cases compared to healthy controls (i.e., −0.51 HC SD, 95% CI: −0.85, −0.17). SST is a growth hormone inhibitory peptide which plays a prominent role in several pathological conditions, including inflammation, diabetes mellitus, Alzheimer’s, Parkinson’s, and acquired immunodeficiency syndrome [45]. In previous studies, SST levels in MS patients’ CSF were significantly decreased compared to healthy controls, and this decrease has been linked to diminished cognitive function and increased cytokine levels [46,47]. Moreover, SST has been linked to control cytokines and cytokine-related pathways, including toll-like receptor4 and nuclear factor-kappa B, which led to the hypothesis that SST might play a role in inflammation inducing damage to the BBB [45]. Considering the results from our sample, plasma SST levels were also decreased in MS cases compared to healthy controls besides CSF findings mentioned previously, leading to the possibility of investigating this potential biomarker in a less invasive way.

CP was lower, 0.38 HC SD (95% CI: −0.64, −0.13), compared to healthy controls, with similar differences found in RRMS cases and SPMS case comparisons with healthy controls. The plasma CP level was highly negatively correlated with SST (*r* = −0.71). CP is an acute phase protein which plays a fundamental role in copper (Cu) and Fe metabolism; it has been shown, in in vivo studies, to be an effective antioxidant in the CNS protecting neural cells against oxidative stress [48]. Our result is in conflict with previous findings which highlighted increased CP serum levels in MS cases compared to healthy controls, attributing this increase to the inability to incorporate CP due to the highly oxidative environment found in the serum of MS patients [49,50]. Nevertheless, decreased serum CP levels have been detected in Parkinson and Alzheimer’s disease cases compared to healthy controls, indicating that this deficiency may contribute to neuronal cell death due to an iron-mediated increase in free radicals [51,52,53]. Therefore, further studies are needed to clarify this unexpected finding.

Lastly, PLAT, or tPa, protein levels in MS cases were estimated on average lower, 0.32 HC SD (95% CI: −0.54, −0.10), as compared to healthy controls, with a higher decrease found in RRMS cases compared to healthy controls (i.e., −0.44 HC SD, 95% CI: −0.72, −0.16). PLAT is a secreted serine protease that converts the proenzyme plasminogen to plasmin, a fibrinolytic enzyme [54]. This protease, expressed in neurons throughout the developing and the adult nervous systems [55], has pleiotropic roles in the CNS including neuronal migration, axonal growth, and synaptic plasticity, as well as a contribution to neurodegeneration in pathological states and the regulation of neurovascular and neuronal response [56,57,58]. PLAT levels in CSF were found to be highly increased in MS cases compared to healthy controls, while, in plasma, PLAT levels were lower in MS cases (but not statistically significantly) [59]. In another study [60] PLAT levels in CSF were instead found to be significantly decreased in SPMS cases compared to healthy controls. Interestingly, and consistently, plasma plasminogen activator inhibitor-1 (PAI-1) levels were found to be significantly higher in MS case controls, specifically RRMS cases, consistently with our finding of a higher plasma PLAT decrease found in RRMS cases compared to healthy controls (as described previously) [61]. An imbalance in the PLAT–PAI-1 ratio has been previously highlighted to play a role in compromising axonal integrity [62]. In fact, decreased PLAT activity, due to increased PAI-1 levels, impairs the capacity of PLAT to clear the fibrinogen deposits (fibrinolysis), at sites of BBB breakdown, demyelinated axons, and inflammatory MS lesions, therefore contributing to axonal damage [63,64]. These results are therefore consistent with the estimated lower plasma PLAT protein levels in MS cases found in our sample, and further highlight the potential risk role played by an imbalanced PLAT–PAI-1 ratio in the neurodegeneration.

In the subsequent analysis, for the identified candidate biomarkers, the variability explained by a set of well-established MS-risk SNPs was quantified, with the aim of improving the functional understanding of GWASs results, helping understanding disease etiology, and gaining more evidence in favor of the identified biomarkers. The analysis was performed on healthy controls only, to avoid MS status exerting potential reverse causation on plasma protein levels. This analysis revealed a significant portion of C9 and CYP24A1 variability explained by, respectively, two and three MS-risk SNPs, i.e., 16% (95% CI: 9%, 39%) for C9 plasma levels and 26% (95% CI: 10%, 44%) for CYP24A1 plasma levels. 

Suggestive additional biomarkers SST, CP, and PLAT plasma level variability were also explored. While plasma SST levels were not significantly associated with any MS-risk SNPs, 16% (95% CI: 6%, 33%) of CP plasma protein level variability was jointly explained by three MS-risk SNPs. Interestingly, almost one-third of PLAT plasma level variability was jointly explained by five MS-risk SNPs, i.e., 32% (95% CI: 13%, 49%). 

In summary, we found four different proteins (C9, CYP24A1, CP, and PLAT), with a potential role in MS susceptibility as highlighted previously from different studies, whose plasma level variability was significantly explained (≥16%) by well-established MS-risk SNPs. Therefore, these results could help explain the possible role of these genetic variants, for which, to date, the causal pathway of disease susceptibility is largely unknown. 

The study presented strengths and limitations. The strength for the first analysis, where plasma protein level differences were evaluated between MS cases and healthy controls, was the high power achieved with our sample size, which made the results more reliable, while potential limitations were represented by: (1) the lack of concomitant treatment data, taken (if any) by MS cases at the time of blood sampling, which could have potentially affected plasma protein levels [19,65], even though the literature on this topic is still not widely explored; (2) the lack of validation of plasma protein level measurements with other methodologies (such as Western blot). The second analysis, where biomarker level variability, explained by a set of well-known MS-risk SNPs, was quantified, was limited by a lower sample size (*N* = 91) compared to the first analysis, which led to a lower power to detect significant signals and potentially leading to inflated effect sizes in significant discoveries (winner’s curse). Nevertheless, unlike GWASs and large-scale QTL analyses, where millions of genetic variants are tested, only a small subset of SNPs were considered and tested. Therefore, this second analysis represented a first exploratory, in a colocalization perspective, attempt to link MS-risk SNPs to MS-related plasma protein levels which could serve as a starting point for further investigations with larger samples.

Together our results represent an important step toward the identification of candidate diagnostic biomarkers in plasma for MS. Even if a future dedicated assay is required to analytically validate our results and to extend them in clinical practice, our study represents the first step in defining the role of circulating proteins towards a rational therapeutic strategy choice, other than increasing the comprehension of the mechanisms underlying the disease. Furthermore, the identification of reliable peripheral, easy to obtain, candidate biomarkers for MS, to be further investigated in forthcoming studies, bears the potential to partially substitute the current invasive lumbar puncture procedure for obtaining CSF and for an improved diagnosis of MS, monitoring the disease activity and progression, and evaluation of treatment responses.

## 4. Materials and Methods

### 4.1. Sample Collection and Genotyping

A register of MS cases diagnosed according to Poser’s criteria [66] was established in Sardinia’s Nuoro province, Italy, in 1995. From this register, data from 20 Sardinian families with 96 MS cases and 437 healthy controls for a total of 533 subjects were available. A genealogical questionnaire was used to reconstruct the pedigrees from grandparents to first-degree cousins. Subjects affected with MS were classified as RRMS or SPMS disease course. 

Genotyping data were obtained using Immunochip [67] from a previous study [68], where the quality control-filtered dataset included 131.497 SNPs.

R Studio, version 1.4.1106, has been used for all analyses.

### 4.2. Measuring Plasma Protein Levels 

Proteins in plasma were studied in a high throughput manner by using the large resource of polyclonal antibodies made available by the HPA project (www.proteinatlas.org/, accessed on 12 July 2021). A total of 56 plasma protein levels were obtained using a bead-based antibody array format, consisting of human platelet antigen antibodies immobilized onto microspheres in suspension. Through a setup that profiles proteins via direct labelling of whole and unfractionated samples with biotin, targets could be analyzed in a multiplexed manner using antibody-conjugated bead arrays of a desired composition with instant data acquisition from the flow cytometer-like Flexmap 3D instrument. To determine relative signal intensities from the binding of antibodies to their target antigens, median fluorescent intensities are displayed when counting at least 50 events per bead ID. The obtained data were processed using probabilistic quotient normalization over the entire dataset to account for possible differences in sample dilution. Finally, 56 antibodies, targeting 56 unique proteins (listed in Supplementary Table S1), selected from suggestions by the clinical collaborators and from proteomic, mRNA, and cDNA expression data found in the literature for their suggested association with MS, both functionally and as a biomarker, were included for the analysis.

### 4.3. Differences in Protein Levels

The first step of our analysis was to search for significant and potentially clinically important differences in protein levels between MS cases and healthy controls. 

Since subjects were related, differences in protein levels between MS cases and healthy controls in the 212 subjects (69 MS cases, 143 healthy controls), having measured protein levels, were evaluated using a linear mixed model (LMM) formulated as:

(1) $$Y_{ij}=\beta_{0}+{\mathrm{MS}}_{ij}*\beta_{1}+{\mathrm{SEX}}_{ij}*\beta_{2}+{\mathrm{AGE}}_{ij}*\beta_{3}+Z_{1i}*{\mathrm{kinship}}_{i}+Z_{2i}*{\mathrm{family}}_{i}+e_{ij}$$

where *j* denotes the individual and *i* the corresponding family, $Y_{ij}$ is the standardized protein level (using mean and standard deviation from healthy control, as explained below), $\beta_{0}$ is the intercept term, ${\mathrm{MS}}_{ij}$ is the MS status (reference = controls) with $\beta_{1}$ the corresponding fixed effect, ${\mathrm{SEX}}_{ij}$ is the sex of the individual (reference = males) with $\beta_{2}$ the corresponding fixed effect, ${\mathrm{AGE}}_{ij}$ is the age of the individual at the day of the blood sampling (obtained as the difference of the day of blood sampling and date of birth) with $\beta_{3}$ the corresponding fixed effect. ${\mathrm{kinship}}_{i}$ was the random effect accounting for a familiar relationship distributed as $N\left(0,\;\sigma^{2}{}_{G}A\right)$, where A was the kinship matrix multiplied by 2 (or the relationship matrix). ${\mathrm{family}}_{i}$ was the random effect accounting for the shared environmental effect with other members of the sub-family effect distributed as $N\left(0,\;\sigma^{2}{}_{C}H\right)$, where H is the matrix with value “1” for the individuals belonging to the same family, and $e_{ij}$ was the residual error assumed to be distributed as $N\left(0,\;\sigma^{2}{}_{I}\right)$. $Z_{1i}$ and $Z_{2i}$ denote the random effect model matrices for ${\mathrm{kinship}}_{i}$ and ${\mathrm{family}}_{i}$. $\sigma^{2}{}_{G}$ and $\sigma^{2}{}_{C}\;$were assumed to be independent. Sex and age at blood sampling were included in the model as fixed effects to avoid potential confounding. The female-to-male MS prevalence ratio in the province of Nuoro (Sardinia, Italy) was reported to be 2:1 [24], a result in line with the worldwide estimate of 2.3–3.5:1 [69]. The association between sex and immune response level was explained in depth in the review by Klein SL and Flanagan [70], where they noted: “sex is one variable that influences innate and adaptive immune responses, resulting in sex-specific outcomes from infectious and autoimmune diseases, malignancies, and vaccines;” this claim was further supported by other subsequent studies [71,72]. Age is also considered a potential confounder for immune protein levels and MS association, as highlighted in a review of neuronal and glial CSF biomarkers in MS [73]; moreover, Lind et al. highlighted the magnitude of plasma proteins changes in adults during a 10-year follow-up, with 61 out of 84 changing significantly [74]. Thus, these insights justified the inclusion of sex and age at blood sampling in the model. relmatLmer function, from lme4qtl R package [75], was used to fit LMM using the maximum likelihood (ML) method. The inference for MS fixed effect $\beta_{1}$ was based on Wald test statistic [76]. 

Since protein levels were rarely normal distributed, but rather right-skewed, using LMM could have led to incorrect inference in the presence of the non-normality of residual distribution. Moreover, an average protein level difference may have masked stronger or weaker differences that may have existed at other points of the distribution. The normality of residuals obtained from LMM was evaluated following guidelines from Kim [32], i.e., in our sample, we rejected the null hypothesis of normality of residuals for an absolute z-value over 3.29 for skewness or excess kurtosis statistics. If the null hypothesis of the normality of residuals assumption was rejected, an LQMM, as formulated by Geraci and Bottai, was used instead [77]. The model is based on quantile regression (QR), a methodology which extends regression for the mean to the analysis of the entire conditional distribution of the outcome variable and does not make assumptions about the model residuals, thus providing a complete picture of the distributional effects using maximum likelihood methods. The model follows the same equation as Equation (1) with the exception that the protein level differences in MS cases and healthy controls were evaluated at the 50th quantile τ (i.e., the median). The 25th and 75th quantiles were also explored for proteins where statistical significance for the median was achieved. The $\beta_{1}$ standard error $se\left(\hat{\beta_{1}}\right)$ estimate was based on block-bootstrap; *B* = 1000 bootstrap samples were obtained by resampling the i=1,…,M sub-families with replacement, and the standard deviation of *B* distribution *sd*(*B*) was used as standard error estimate $se\left(\hat{\beta_{1}}\right)$. Parametric 95% confidence intervals are reported based on the t-value statistic. [78]. The lqmm function from lqmm R package [79] was used to fit LQMM. Additional statistical details on LQMM can be found in Supplementary Materials S1.

Both LMM and LQMM were fitted on centered and standardized protein levels, using the mean and SD of protein levels in healthy controls, to get a better interpretability of the estimated coefficients. Thus, for a specific protein, an estimated coefficient $\beta_{1}$ would translate as an increase/decrease in MS case protein level (mean or median, depending on the model used) equal to $\beta_{1}$ times the SD of the protein levels in healthy controls. This interpretation gave direct and more understandable evidence of the magnitude of protein level differences between MS cases and healthy controls. 

Once *p*-values were obtained both for LMM or LQMM, these were corrected to avoid type I error inflation due to multiple testing (i.e., 56 statistical tests, one for each protein). The Holm procedure was used to provide strong control on FWER at the 0.05 level since it does not require the independence of the test statistics [80], which we could not assume since proteins were correlated both positively and negatively. Plasma protein differences between MS cases and healthy controls, which did not reach statistical significance but showed a *p*-value < 0.005 and an absolute difference of at least 0.3 HC SD, were still considered potentially interesting proteins to be investigated. For these proteins, both significant and “suggestive,” Pearson’s correlation coefficients were calculated.

Finally, comparisons for plasma protein levels significant after multiple testing corrections were explored within MS course classifications, i.e., protein level differences were tested between RRMS cases and SPMS cases compared to healthy controls, as well as between SPMS cases and RRMS cases, using the same model in Equation (1).

### 4.4. Power Calculation

Prior to the evaluation of protein level differences on our sample, we estimated the accuracy of the methods described in Section 4.3. applied on our sample size and its pedigree structure. Therefore, the power to detect a fixed $\beta_{1}$ effect = 0.3 and 0.5 at significance level α = 0.05 was evaluated using both LMM and LQMM. The same pedigree from our data was used to simulate protein levels. For LMM, the parameters were fixed as follows (all parameters are explained in Equation (1)):

(2) $$\begin{array}{c} \beta_{0}\;=0\\ \beta_{1}=0.3,\;0.5\\ \beta_{2}=0.05\\ \beta_{3}=0.01\\ \sigma^{2}{}_{G}=0.15\\ \sigma^{2}{}_{C}=0.10\\ \sigma^{2}{}_{I}\;=1-(\sigma^{2}{}_{G}\;+\sigma^{2}{}_{C}\;+var\left(\beta_{1}*\mathrm{MS}+\beta_{2}*\mathrm{SEX}+\beta_{3}*\mathrm{AGE}\right)) \end{array}$$

β effects were in the scale of plasma protein levels SD as measured in healthy controls (HC SD), e.g., for β = 0.5 the plasma protein levels in MS cases were higher by a half SD as measured in healthy controls. The power to detect a plasma protein level difference of 0.5 HC SD is of interest because this value has been arbitrarily selected to represent the MCID, which is defined as the smallest the change in score of a patient-reported outcome (either beneficial or harmful) that is important from the patient or clinician’s perspective [31]. Even if this measure is used to evaluate treatment efficacy, we used it as a reference value to highlight a potentially meaningful physiological change. The power to detect a plasma level difference of 0.3 HC SD has been evaluated to verify our power to detect lower plasma protein levels differences.

Sex and age at blood sampling were already defined based on our pedigree, and their fixed effects $\beta_{2}$ and $\beta_{3}$ fixed to 0.05 and 0.01, i.e., female/male protein level differences have been simulated as 0.05 HC SD and each age unit increase (in years) has been associated with a protein level increase by 0.01 HC SD (or, equally, an increase in 10 years in age has been associated with a protein level increase of 0.10 HC SD). Since an estimate of age and sex effects on each plasma protein level could not be found in the literature, and the effects could differ between proteins, we have fixed, a priori, small β effects. Regardless, sex and age effects are expected to have a minor influence on power evaluation compared to the impact of the sample size alone. 

MS was simulated as a random Bernoulli variable, i.e., MS~Bernoulli(0.325), where the parameter *p* = 0.325 is the actual proportion of MS cases in our data.

kinship and family random effects (kinship and shared environment effects) were simulated as a random multivariate normal variable, i.e., $\mathrm{kinship}\sim MVN(0,\;\sigma^{2}{}_{G}A$), $\mathrm{family}\sim MVN(0,\;\sigma^{2}{}_{C}H$). We chose a 0.15 value for $\sigma^{2}{}_{G}$ and 0.10 for $\sigma^{2}{}_{C}$ following the results from Liu et al. [81], which estimated the mean proportion of heritability and shared environment effect for 372 plasma protein levels.

Residuals e were generated from a random normal distribution, i.e., $e\sim N(0,\;\sigma^{2}{}_{I}$), where $\sigma^{2}{}_{I}$ was simply calculated as the variance component that sums to 1 after considering the sum of $\sigma^{2}{}_{G}$_,_ $\sigma^{2}{}_{C}$ and the variance of the fixed-effects component (i.e., $var\left(\beta_{1}*\mathrm{MS}+\beta_{2}*\mathrm{SEX}+\beta_{3}*\mathrm{AGE}\right)$). Protein levels were then generated 2000 times using the formula in Equation (1). The power to detect effect sizes $\beta_{1}$ = 0.3 and 0.5 was evaluated at α = 0.05 using LMM. 

Simulations to evaluate power using the LQMM model were conducted fixing the same parameters as the previous LMM simulation except for residuals e; these were generated from a random asymmetric Laplace distribution ALD(0, σ, τ) with scale parameter σ fixed to 0.2 and τ, which stands for the respective quantile to be estimated, fixed to 0.5. In each LQMM simulation, $se\left(\hat{\beta_{1}}\right)$ was estimated using 50 block-bootstrap replications (as defined in Section 4.3.).

### 4.5. MS-Risk SNPs-Protein Levels Associations

The second step of our analysis was to quantify plasma protein level variability explained by a set of well-known MS-risk SNPs. This analysis, performed on the protein levels, was significantly different between MS cases and healthy controls in the previous step of the analysis only. The list of MS-risk SNPs was obtained from [27], where 200 autosomal SNPs outside the major histocompatibility complex (MHC) region were prioritized as significantly and strongly suggestive of being associated with MS risk. From these 200 prioritized signals only 139 SNPs could be selected for our analysis as included in our ImmunoChip data. 

This “naïve” approach did not have any intention to establish causality between protein levels and MS but represents solely an attempt to investigate the potential biological function of well-established MS-risk SNPs for which, to date, the causal pathway is still unknown.

The analysis was conducted on the 92 healthy controls having both proteins and ImmunoChip data. We excluded MS cases from the analysis since the aim was to obtain SNP–protein associations in regular healthy conditions, avoiding potential reverse causation due to a different physiological status caused by the disease. To avoid a lack of precision and/or type I error inflation due to a reduced sample size, we kept in our sample only variants having MAF > 0.10. Moreover, variants in linkage disequilibrium, considering a maximum threshold for the $r^{2}$ statistic equal to 0.2, were also removed. This caused the removal of 16 and 4 variants, respectively, leading to 119 SNPs being included in the analysis.

Since all 119 MS-risk SNPs could not be included in a single model, as parameters would outnumber observations, we first refined the search for MS-risk SNPs potentially associated with protein levels following the approach in [82,83]. First, each SNP was included as a covariate in a univariate LMM formulated as:

(3) $$Y_{ij}=\beta_{0}+{\mathrm{SEX}}_{ij}*\beta_{1}+{\mathrm{AGE}}_{ij}*\beta_{2}+{\mathrm{SNP}}_{ij}*\beta_{3}+Z_{1i}*{\mathrm{kinship}}_{i}+Z_{2i}*{\mathrm{family}}_{i}+e_{ij}$$

where all the variables were already defined in Equation (1) except for ${\mathrm{SNP}}_{ij}$, which denotes the number of effect alleles (minor allele), with $\beta_{1}$ the respective additive linear effect on protein levels. Among the SNP–protein level associations significant at α = 0.10, the best set of SNP markers were selected using the stepwise regression procedure [84], where the inclusion and exclusion of each SNP of the model was determined at the 0.05 level. The best set of SNPs was then included in a multivariable LMM model, formulated as in Equation (3), and SNPs significantly associated with protein levels at α = 0.01 were selected to estimate the marginal proportion of protein level variability explained by significant SNPs. This measure has been calculated using the marginal *R^2^* statistic as defined by Nakagawa and Schielzeth [85]:

(4) $$R^{2}{}_{\mathrm{SNPs}}=\frac{\sigma^{2}{}_{\mathrm{SNPs}}}{\sigma^{2}{}_{F}+\sigma^{2}{}_{G}+\sigma^{2}{}_{C}+\sigma^{2}{}_{I}}$$

where $\sigma^{2}{}_{I}$, $\sigma^{2}{}_{C}$, $\sigma^{2}{}_{C}$ were defined as in Equation (1), $\sigma^{2}{}_{F}$ was the variance for the fixed effects components (i.e., sex, age at blood sampling, and the set of SNPs included in the multivariate model) and $\sigma^{2}{}_{\mathrm{SNPs}}$ was the variance for the significant SNPs, at α = 0.01, the fixed effects component. A marginal *R^2^*_SNP_ statistic was also calculated separately for each significant SNP (the sum of each *R^2^*_SNP_ giving *R^2^*_SNPs_). More details are provided in Supplementary Materials S1. A 95% confidence interval for $R^{2}{}_{\mathrm{SNPs}}$ was calculated making use of BCa interval, at α = 0.05, calculated on *B* = 1000 block-bootstrap replications (as defined in Section 4.3) [78].

## Figures and Tables

**Table 1:** Sample descriptive statistics for 212 subjects having protein levels.

			Age at Blood Sampling		Age at MS Onset	
	*n*	% Females	Median	IQR ^1^	Median	IQR ^1^
Number of subjects	212	54.7%	49.3	36.0–64.5		
MS cases	69 (32.5%)	66.7%	39.3 ^3^	32.5–48.2	27.0 ^4^	21.0–34.0
RRMS ^5^	41 (59.4%) ^2^	73.2%	34.9	29.4–42.4	26.0	20.0–34.0
SPMS ^6^	18 (26.1%) ^2^	61.1%	45.7	42.2–50.8	29.0	24.5–37.0
Not reported	10 (14.5%) ^2^	50.0%	41.2 ^3^	36.1–45.9	23.0 ^4^	22.8–29.0
Healthy controls	143 (67.4%)	49.0%	57.2 ^3^	40.5–69.0		

^1^ Interquartile Range; ^2^ Percentages are referred to the total of MS cases; ^3^ 3 subjects had missing age at sampling; ^4^ 6 subjects had missing age at onset; ^5^ RRMS = Relapse-Remitting MS; ^6^ SPMS = Secondary Progressive MS.

**Table 2:** Significant and suggestive protein level differences between MS cases and healthy controls.

Protein	Chromosome	HPA ^1^	Model ^2^	Estimate ^3^	SE ^4^	95% CI ^5^	Raw *p*-Value ^6^	Corrected *p*-Value ^7^
C9	5	029577	LMM	0.529	0.143	[0.248, 0.810]	<0.001	0.012
CYP24A1	20	022261	LQMM	0.418	0.116	[0.188, 0.647]	<0.001	0.023
SST	3	019472	LQMM	−0.371	0.126	[−0.619, −0.123]	0.004	0.190
CP	3	001834	LQMM	−0.381	0.130	[−0.638, −0.125]	0.004	0.195
PLAT	8	003412	LMM	−0.322	0.113	[−0.543, −0.101]	0.004	0.217

^1^ Human Protein Atlas antibody product name (Human Platelet Antigen); ^2^ LMM = linear mixed model, LQMM = linear quantile mixed model; ^3^ difference between MS cases and healthy controls expressed as number of standard deviations in healthy controls. Estimate is based on the mean for LMM models and on the median for LQMM models; ^4^ standard error; ^5^ confidence interval; ^6^ adjusted for sex, age at blood sampling day, kinship effect, and shared environment effect; ^7^ corrected, due to multiple testing, using Holm procedure.

**Table 3:** 25th and 75th quantile estimates for protein level differences between MS cases and healthy controls in proteins resulted statistically significant at 50th quantile after multiple testing correction.

Protein	Quantile	Estimate ^1^	SE ^2^	95% CI ^3^	*p*-Value ^4^
CYP24A1	25th	0.413	0.117	[0.182, 0.643]	<0.001
CYP24A1	75th	0.418	0.116	[0.188, 0.647]	<0.001
SST	25th	−0.391	0.126	[−0.639, −0.143]	0.002
SST	75th	−0.371	0.126	[−0.621, −0.122]	0.004
CP	25th	−0.386	0.130	[−0.643, −0.129]	0.003
CP	75th	−0.379	0.130	[−0.635, −0.122]	0.004

^1^ Difference between MS cases and healthy controls expressed as number of standard deviations in healthy controls; ^2^ standard error; ^3^ confidence interval; ^4^ adjusted for sex, age at blood sampling day, kinship effect, and shared environment effect.

**Table 4:** Two-group comparison within MS course classifications for statistically significant and suggestive plasma biomarkers.

Protein	Chromosome	HPA ^1^	Model ^2^	Comparison	Estimate ^3^	SE ^4^	95% CI ^5^	*p*-Value ^6^
C9	5	029577	LMM	RRMS vs. HC	0.441	0.174	[0.100, 0.782]	0.011
			LMM	SPMS vs. HC	0.624	0.229	[0.176, 1.073]	0.006
			LQMM	SPMS vs. RRMS	0.187	0.308	[−0.431, 0.807]	0.545
CYP24A1	20	022261	LQMM	RRMS vs. HC	0.457	0.156	[0.151, 0.766]	0.004
			LQMM	SPMS vs. HC	0.317	0.217	[−0.111, 0.746]	0.146
			LMM	RRMS vs. SPMS	−0.090	0.319	[−0.716, 0.536]	0.778
SST	3	019472	LQMM	RRMS vs. HC	−0.284	0.152	[−0.583, 0.015]	0.063
			LQMM	SPMS vs. HC	−0.507	0.173	[−0.849, −0.166]	0.004
			LQMM	RRMS vs. SPMS	−0.065	0.201	[−0.468, 0.338]	0.748
CP	3	001834	LQMM	RRMS vs. HC	−0.281	0.177	[−0.630, 0.068]	0.114
			LQMM	SPMS vs. HC	−0.395	0.141	[−0.673, −0.117]	0.006
			LQMM	RRMS vs. SPMS	0.011	0.255	[−0.501, 0.523]	0.967
PLAT	8	003412	LMM	RRMS vs. HC	−0.441	0.144	[−0.723, −0.159]	0.002
			LMM	SPMS vs. HC	−0.346	0.182	[−0.704, 0.012]	0.058
			LMM	RRMS vs. SPMS	0.221	0.321	[−0.407, 0.850]	0.491

^1^ Human Protein Atlas antibody product name (human platelet antigen); ^2^ LMM = linear mixed model, LQMM = linear quantile mixed model; ^3^ For RRMS vs. HC and SPMS vs. HC comparison: difference between MS cases and healthy controls expressed as number of standard deviations in healthy controls. For SPMS vs. RRMS: difference between SPMS cases and RRMS expressed as number of standard deviations in RRMS cases. Estimate is based on the mean for LMM models and on the median for LQMM models; ^4^ standard error; ^5^ confidence interval; ^6^ adjusted for sex, age at blood sampling day, kinship effect, and shared environment effect; RRMS = relapse-remitting MS; SPMS = secondary progressive MS; HC = healthy controls.

**Table 5:** Marginal proportion of protein level variability explained by significant MS-risk SNP allele additive effect.

Protein	Chr ^1^	HPA ^2^	N° SNPs ^3^	SNP ^4^	SNP Position (chr:bp) ^5^	Effect Allele ^6^	MAF ^7^	Additive Effect ^8^	SE ^9^	*p*-Value ^10^	*R* ^2^_SNP_^11^	*R* ^2^_SNPs_^12^	95% CI ^13^
C9	5	029577	5	rs2104286	10:6099045	C (T)	0.19	0.61	0.15	<0.001	0.10	0.16	[0.09–0.39]
				rs1610555	18:67543147	T (G)	0.32	0.32	0.12	0.009	0.06		
CYP24A1	20	022261	4	rs1870071	19:16505106	C (T)	0.21	0.64	0.16	<0.001	0.12	0.26	[0.10–0.44]
				rs11567694	5:35857704	G (A)	0.23	0.55	0.17	<0.001	0.09		
				rs7665090	4:103551603	G (A)	0.46	−0.35	0.12	0.004	0.05		
SST	3	019472	2	-	-	-	-	-	-	-	-	-	-
CP	3	001834	5	rs2082881	2:25038268	G (A)	0.24	0.43	0.15	0.004	0.06	0.16	[0.06–0.33]
				rs756699	5:133446575	C (T)	0.13	0.54	0.20	0.006	0.05		
				rs17886724	17:40496163	G (A)	0.35	−0.32	0.12	0.009	0.05		
PLAT	8	003412	8	rs11567694	5:35857704	G (A)	0.23	0.63	0.14	<0.001	0.11	0.32	
				rs3766374	1:160720554	A (G)	0.21	−0.49	0.14	<0.001	0.08		
				rs12086448	1:160393905	G (A)	0.43	0.39	0.11	<0.001	0.06		[0.13–0.49]
				rs212397	6:159474624	C (A)	0.27	−0.32	0.12	0.008	0.05		
				rs9947399	18:56271544	G (A)	0.47	0.29	0.11	0.009	0.04		

^1^ Chromosome position of coding gene; ^2^ Human Protein Atlas antibody product name (human platelet antigen); ^3^ number of SNPs, selected in the stepwise procedure, included in the multivariable SNP–protein level model; ^4^ SNPs significantly associated with protein levels at α = 0.01 in the multivariable model; ^5^ SNP position based on human genome 19; ^6^ the effect allele is represented by the minor allele in our ImmunoChip data. The allele between brackets is the reference allele; ^7^ minor allele frequency; ^8^ additive effect due to one effect allele on protein levels resulting from the multivariable SNP–protein level model (controlling for sex, age, kinship effect, shared environment effect, and the other SNPs included in the multivariable model); ^9^ standard error; ^10^ *p*-value for null hypothesis of additive effect equal to 0; ^11^ proportion of protein levels variability explained by the additive effect of the specific significant SNP; ^12^ proportion of protein levels variability jointly explained by additive effects of significant SNPs at α = 0.01; ^13^ 95% confidence interval.

## Data Availability

Raw data was uploaded as Supplementary Materials.

## Supplementary Materials

The following are available online at https://www.mdpi.com/article/10.3390/life12020151/s1, Supplementary Table S1: List of plasma proteins measured; Supplementary Table S2: Plasma proteins differences between MS cases and healthy controls from LMM/LQMM models; Supplementary Table S3: SNPs-protein levels associations from univariate and multivariate LMM models; data_sardinian: Dataset used for the analyses, comprising pedigree, age, disease course, protein levels and genotyping information; R Codes: Folder with R codes used for all analyses; Details of statistical analysis are reported in Supplementary Materials S1.
