# Supplementary material for: Plasma Protein Levels Analysis in Multiple Sclerosis Sardinian Families Identified C9 and CYP24A1 as Candidate Biomarkers
Source: Life (Basel). 2022 Jan 20;12(2):151. doi: 10.3390/life12020151 (PMC8879906; doi:10.3390/life12020151)
Supplement: Supplementary file 1 [file life-12-00151-s001.zip › life-1513114-supplementary/Supplementary Materials/Supplementary Materials S1.pdf]

life

Supplementary Materials S1

4.3. Measuring plasma protein levels

The first step of our analysis was to search for significant and potential clinical important differences in protein levels between Multiple Sclerosis (MS) cases and healthy controls.

Since subjects were related, differences in protein levels between MS cases and healthy controls in the 212 subjects (69 MS cases, 143 healthy controls) having protein levels measured were evaluated using a Linear Mixed Model (LMM) formulated as:

$$Y_{ij} = \beta_0 + MS_{ij} * \beta_1 + Sex_{ij} * \beta_2 + Age_{ij} * \beta_3 + Z_{1i} * kinship_i + Z_{2i} * family_i + e_{ij} \qquad Eq\ (1)$$

Where j denoted the individual and i the corresponding family, Y_{ij} was the standardized protein level (using mean and standard deviation from healthy control, as explained below), β_0 was the intercept term, MS_{ij} was the MS status (reference=controls) with β_1 the corresponding fixed effect, $Sex_{ij}$was the sex of the individual (reference=males) with β_2 the corresponding fixed effect, Age_{ij} was the age of the individual at the day of the blood sampling (obtained as the difference of the day of blood sampling and date of birth) with β_3 the corresponding fixed effect. $kinship_i$ was the random effect accounting for familiar relationship distributed as $N(0, \sigma^2{}_G A)$, where A was the kinship matrix multiplied by 2 (or relationship matrix). $family_i$ was the random effect accounting for the shared environmental effect with other members of the sub-family effect distributed as $N(0, \sigma^2{}_C H)$, where H is the matrix with value "1" for the individuals belonging to the same family, and e_{ij} was the residual error assumed to be distributed as $N(0, \sigma^2{}_I)$. Z_{1i} and Z_{2i} denoted the random effect model matrices for $kinship_i$ and $family_i$. $\sigma^2{}_G$ and $\sigma^2{}_C$ were assumed to be independent. Sex and age at blood sampling were included in the model as fixed effects to avoid potential confounding. The female-to-male MS prevalence ratio in the province of Nuoro (Sardinia, Italy) was reported to be 2:1 [1] a result in line with the worldwide estimate of 2.3–3.5:1 [2]. The association between sex and immune response level was explained in depth in the review by Klein SL & Flanagan [2], where they noted: "sex is one variable that influences innate and adaptive immune responses, resulting in sex-specific outcomes from infectious and autoimmune diseases, malignancies, and vaccines"; this claim was further supported by other subsequent studies [3-5]. Age is also considered a potential confounder for immune protein levels and MS association, as highlighted in a review of neuronal and glial cerebrospinal fluid (CSF) biomarkers in MS [6]; moreover, Lind et al. highlighted the magnitude of plasma proteins changes in adults during a 10-year follow-up, with 61 out of 84 changing significantly [7]. Thus, these insights justified the inclusion of sex and age at blood sampling in the model. *relmatLmer* function, from *lme4qtl* R package [8], was used to fit LMM using Maximum Likelihood (ML) method. Inference for MS fixed effect β_1 was based on Wald test statistic [9].

Since protein levels were rarely normal distributed, but rather right-skewed, using LMM could have led to incorrect inference in presence of non-normality of residuals distribution. Moreover, an average protein level difference may have masked stronger or weaker differences that may have existed at other points of the distribution. Normality of residuals obtained from LMM was evaluated following guidelines from Kim [10], i.e., in our sample, we rejected the null hypothesis of normality of residuals for an absolute z-value over 3.29 for skewness or excess kurtosis statistics. If the null hypothesis of normality of residuals assumption was rejected, a Linear Quantile Mixed Model (LQMM), as formulated by Geraci and Bottai, was used instead [11]. The model is based on Quantile Regression (QR), a methodology which extends regression for the mean to the analysis of the entire conditional distribution of the outcome variable and does not make assumptions about the model residuals, thus providing a complete picture of the distributional effects using maximum likelihood methods. The methodology relies on the Asymmetric

Laplace (AL) distribution which allows to estimate the τth conditional quantile using maximum likelihood methods. Considering for the dependent variable y_{ij} a vector $i = 1, \ldots, M$ (where M = number of sub-families, and $j = 1, \ldots, n_i$ with $N = \sum_i n_i$ number of subjects) which values are independently distributed conditional on q random effects vectors u_i to an unknown distribution $F_{y_i|u_i}$, a joint AL model for $y_i|\mu_i^{(\tau)}$ is formulated as $y_i = \mu_i^{(\tau)} + \varepsilon_i^{(\tau)}$, where $\mu_i^{(\tau)} = X_i\beta_x^{(\tau)} + Z_i u_i$ and residuals are distributed following an AL distribution, i.e., $\varepsilon_i^{(\tau)} \sim AL\,(0, \sigma^{(\tau)}, \tau)$. Residuals are independent from the random effect vector u_i, which has median equal to 0. β_x denotes a vector of unknown fixed effects of independent variables X_i and τ indicates the skewness parameter, set a priori, defining the quantile level to be estimated. Finally, even if $F_{y_i|u_i}$ distribution is unknown, its τth quantile is estimated making use of the AL distribution where $\mu^{(\tau)}$, $\sigma^{(\tau)}$ and τ denote the location, scale, and skewness parameters. Adopting the assumption of Laplace distribution for random effects (i.e., $kinship_i \sim Laplace(0, \sigma^2{}_G A)$ and $famid_i \sim Laplace(0, \sigma^2{}_G H)$) directly results into a Gauss–Laguerre quadrature for the approximate AL-based log-likelihood:

$$l_{app}(\beta_x, \sigma, \Psi \mid y) = \sum_{i}^{M} log\left\{\sum_{k_1}^{K} \ldots \sum_{k_q}^{K} p(y_i|\beta_x, \sigma, \Psi' \,\mathrm{v}_{k_1}, \ldots., \mathrm{v}_{k_q}) \prod_{l=1}^{q} \omega_{k_l}\right\}$$

Where the constant K is an integer giving the number of points for each of the q one-dimensional integrals over the real line, $\mathrm{v}_{k_1}, \ldots., \mathrm{v}_{k_q}$=($\mathrm{v}_{k_1}, \ldots., \mathrm{v}_{k_q})^T$ are the nodes and ω_{k_l}, with $l = 1, \ldots \ldots q$ (i.e. the number of random effects), the kernel function based weights. Ψ denotes the diagonal covariance matrix of the random effects. *lqmm* R function does not allow to directly model kinship matrix in the covariance structure, therefore the model matrix Z_i for *kinship*$_i$ random effect has been multiplied by the Cholesky decomposition L of the relationship matrix A (i.e., $A = LL'$). The approach has been described by Harville and Callanan [12] and implemented by Vazquez in *pedigreemm* R package for heritability estimation using pedigrees [13]. The gradient search algorithm for Laplace likelihood has been used to minimize the negative integrated log-likelihood [14].

The protein levels difference in MS cases and healthy controls were evaluated at 50th quantile τ (i.e., the median), using the same model as above. 25th and 75th were also explored for proteins where statistical significance for the median was achieved.

lqmm function from *lqmm* R package [15] was used to fit LQMM, using the default number of quadrature knots (i.e., 7) and the default optimization algorithm based on the gradient of the Laplace log–likelihood. Inference for MS fixed-effect β_1 is based on two-sided t-test with $N - P - 1$ degrees of freedom, where N is the number of subjects in the analysis, and P is the number of parameters in the model (i.e., 7: β_0, β_1, β_2, β_3, $\sigma^{(\tau)}$, $\sigma^2{}_G$, $\sigma^2{}_C$).

T-test statistic T_0 is then calculated as $T_0 = \frac{\widehat{\beta_1}}{se(\widehat{\beta_1})}$.

Where β_1 standard error $se(\widehat{\beta_1})$ estimate was based on block-bootstrap; B=1000 bootstrap samples were obtained by resampling the $i = 1, \ldots, M$ sub-families with replacement, and standard deviation of B distribution $sd(B)$ was used as standard error estimate $se(\widehat{\beta_1})$. Parametric 95% confidence intervals are reported based on t-value statistic.

Bias in $\widehat{\beta_1}$ statistic estimate was estimated as $\widehat{Bias}_B(\widehat{\beta_1}) = \bar{B} - \widehat{\beta_1}$, where $\bar{B}$ was the mean of B distribution. Following Efron and Tibshirani [16], it was suggested and justified that if bias $\widehat{Bias}_B(\widehat{\beta_1}) < 0.25 * se(\widehat{\beta_1})$ then the bias can be ignored. Otherwise, bias correction is implemented to $\widehat{\beta_1}$, subtracting $\widehat{Bias}_B(\widehat{\beta_1})$ estimate (i.e., bias-corrected estimate $\breve{\beta_1} = \widehat{\beta_1} - \widehat{Bias}_B(\widehat{\beta_{1\tau}})$) and t-value statistic,

$T_0 = \frac{\breve{\beta_1}}{se(\widehat{\beta_1})}$, was calculated using the bias-corrected estimate $\breve{\beta_1}$.

Both LMM and LQMM were fitted on centered and standardized protein levels, using the mean and SD of protein levels in healthy controls, to get a better interpretability

of the estimated coefficients. Thus, for a specific protein, an estimated coefficient β_1 would translate as an increase/decrease in MS cases protein level (mean or median, depending on the model used) equal to β_1 times the SD of the protein levels in healthy controls. This interpretation gave direct and more understandable evidence on the magnitude of protein levels difference between MS cases and healthy controls.

Once p-values were obtained both for LMM or LQMM, these were corrected to avoid type I error inflation due to multiple testing (i.e., 56 statistical tests, one for each protein). Holm correction was used to provide strong control on family-wise error (FWER) at 0.05 level since it does not require the independence of the test statistics [17], which we could not assume since proteins were correlated both positively and negatively. Plasma proteins differences between MS cases and healthy controls, which did not reach statistical significance but showed a p-value < 0.005 and an absolute difference of at least 0.3 healthy controls protein plasma levels standard deviations (HC SD), were still considered as potentially interesting proteins to be investigated. For these proteins, both significant and "suggestive", Pearson's correlation coefficients were calculated.

Finally, comparisons for plasma protein levels significant after multiple testing corrections, were explored within MS course classifications, i.e., protein levels differences were tested between Relapse-Remitting MS (RRMS) cases and Secondary Progressive (SPMS) cases compared to healthy controls, as well as between SPMS cases and RRMS cases, using the same model in Eq 1.

4.5. **MS-risk SNPs-protein levels associations**

The second step of our analysis was to quantify plasma protein levels variability explained by a set of well-known MS-risk SNPs. This analysis was performed on the protein levels resulted significantly different between MS cases and healthy controls in the previous step of the analysis only. The list of MS-risk SNPs was obtained from [18], where 200 autosomal SNPs outside the major histocompatibility complex (MHC) region were prioritized as significantly and strongly suggestive of being associated with MS risk. From these prioritized 200 signals 139 SNPs only could be selected for our analysis as included in our ImmunoChip data.

This "naïve" approach had not any purpose to establish causality between protein levels and MS, but it solely represents an attempt to investigate the potential biological function of well-established MS-risk SNPs for which, to date, the causal pathway is still unknown.

The analysis was conducted on the 92 healthy controls having both proteins and ImmunoChip data. We excluded MS cases from the analysis since the aim was to obtain SNP-protein associations in regular healthy conditions, avoiding potential reverse causation due to a different physiological status caused by the disease. To avoid lack of precision and/or type I error inflation due to reduced sample size, we kept in our sample only variants having minor allele frequency (MAF) > 0.10. Moreover, variants in linkage disequilibrium, considering a maximum threshold for r^2 statistic equal to 0.2, were also removed. This caused the removal of 16 and 4 variants respectively, leading to 119 SNPs included in the analysis.

Since all 119 MS-risk SNPs could not be included in a single model, as parameters would outnumber observations, we first refined the search for MS-risk SNPs potentially associated with protein levels following the approach in [19] and [20]. First, each SNP was included as a covariate in a univariate LMM formulated as:

$$Y_{ij} = \beta_0 + SEX_{ij} * \beta_1 + Age_{ij} * \beta_2 + SNP_{ij} * \beta_3 + Z_{1i} * kinship_i + Z_{2i} * family_i + e_{ij} \qquad Eq\ (2)$$

Where all the variables were already defined in Eq 1 except for SNP_{ij}, which denotes the number of effect alleles (minor allele), with β_1 the respective additive linear effect on protein levels. Among the SNPs-protein levels associations significant at α=0.10, the best set of SNP markers were selected using the stepwise regression procedure [21], where inclusion and exclusion of each SNP out of the model was determined at 0.05 level. The

best set of SNPs was then included in a multivariable LMM model, formulated as in Eq 2, and SNPs significantly associated with protein levels at α=0.01 were selected to estimate the marginal proportion of protein level variability explained by significant SNPs. This measure has been calculated using the marginal R^2 statistic as defined by Nakagawa and Schielzeth [22]:

$$R^2_{SNPs} = \frac{\sigma^2_{SNPs}}{\sigma^2_F + \sigma^2_G + \sigma^2_C + \sigma^2_I}$$

Where σ^2_I, σ^2_C, σ^2_C were defined as in Eq 1, σ^2_F was the variance for the fixed effects components (i.e., sex, age at blood sampling, and the set of SNPs included in the multivariate model). In the scenario where all SNPs are significant at α=0.01, this component is defined as:

$$\begin{aligned}\sigma^2_F &= var\left(\beta_1 * SEX_{ij} + \beta_2 * AGE_{ij} + \sum_{h=1}^{p} \beta_h * SNP_{hij}\right)\\ &= var(\sigma^2_{SNPs}) + var(\sigma^2_{SEX,AGE}) + 2 * cov(\sigma^2_{SNPs}, \sigma^2_{SEX,AGE})\\ &= var\left(\sum_{h=1}^{p} \beta_h * SNP_{hij}\right) + var(\beta_1 * SEX_{ij} + \beta_2 * AGE_{ij}) + 2\\ &\quad * cov(\sum_{h=1}^{p} \beta_h * SNP_{hij}, \beta_1 * SEX_{ij} + \beta_2 * AGE_{ij})\end{aligned}$$

Where β_1, β_2, SEX_{ij} and AGE_{ij} were defined as in equation (1), β_h were the significant SNPs fixed effects, with $h = 1, \dots, p$ denoting the specific SNP. The covariance component $cov(\sum_{h=1}^{p} \beta_h * SNP_{hij}, \beta_1 * SEX_{ij} + \beta_2 * AGE_{ij})$ results different from 0 (with positive or negative values) when sex and age at blood sampling are not independent from the set of significant SNPs and therefore jointly shared a part of the information about σ^2_F (and consequently about protein levels). σ^2_{SNPs} and $\sigma^2_{SEX,AGE}$, were, respectively, the variance for the SNPs significant at α=0.01 fixed effects component and the variance for joint sex and age at the blood sampling fixed effects component. These were then defined as:

$$\sigma^2_{SNPs} = var\left(\sum_{h=1}^{p} \beta_h * SNP_{hij}\right) + cov(\sum_{h=1}^{p} \beta_h * SNP_{hij}, \beta_1 * SEX_{ij} + \beta_2 * AGE_{ij})$$

$$\sigma^2_{SEX,AGE} = var(\beta_1 * SEX_{ij} + \beta_2 * AGE_{ij}) + cov(\sum_{h=1}^{p} \beta_h * SNP_{hij}, \beta_1 * SEX_{ij} + \beta_2 * AGE_{ij},)$$

Where the sum of σ^2_{SNPs} and $\sigma^2_{SEX,AGE}$, gives σ^2_F. In case of non-significant SNPs resulting from the multivariable model, at α=0.01, these were added to $\sigma^2_{SEX,AGE}$, component.

Marginal R^2_{SNPs} statistic was also calculated separately for each significant SNP, (the sum of each R^2_{SNP} giving R^2_{SNPs}). 95% confidence interval for R^2_{SNPs} was calculated making use of (bias-corrected accelerated) BCa interval, at α=0.05, calculated on B=1000 block-bootstrap replications (as defined in section 4.3) [16].

References

1. Urru, S.A.M.; Antonelli, A.; Sechi, G.M. Prevalence of multiple sclerosis in Sardinia: A systematic cross-sectional multi-source survey. *Mult. Scler. J.* **2020**, *26*, 372– 380, doi:10.1177/1352458519828600.
2. Harbo, H.F.; Gold, R.; Tintora, M. Sex and gender issues in multiple sclerosis. *Ther. Adv. Neurol. Disord.* **2013**, *6*, 237–48, doi:10.1177/1756285613488434.
3. Klein, S.L.; Flanagan, K.L. Sex differences in immune responses. *Nat. Rev. Immunol.* **2016**, *16*, 626–638, doi:10.1038/nri.2016.90.

4. Ortona, E.; Pierdominici, M.; Rider, V. Editorial: Sex hormones and gender differences in immune responses. *Front. Immunol.* **2019**, *10*, 1076, doi:10.3389/fimmu.2019.01076.
5. Morris, G.P. Understanding sex-related differences in immune responses. *Sci. Transl. Med.* **2020**, *12*, doi:10.1126/scitranslmed.abd3631.
6. Momtazmanesh, S.; Shobeiri, P.; Saghazadeh, A.; Teunissen, C.E.; Burman, J.; Szalardy, L.; Klivenyi, P.; Bartos, A.; Fernandes, A.; Rezaei, N. Neuronal and glial CSF biomarkers in multiple sclerosis: a systematic review and meta-analysis. *Rev. Neurosci.* **2021**, *32*, 573–595, doi:10.1515/REVNEURO-2020-0145.
7. Lind, L.; Sundström, J.; Larsson, A.; Lampa, E.; Ärnlöv, J.; Ingelsson, E. Longitudinal effects of aging on plasma proteins levels in older adults - associations with kidney function and hemoglobin levels. *PLoS One* **2019**, *14*, doi:10.1371/JOURNAL.PONE.0212060.
8. Ziyatdinov, A.; Vázquez-Santiago, M.; Brunel, H.; Martinez-Perez, A.; Aschard, H.; Soria, J.M. lme4qtl: linear mixed models with flexible covariance structure for genetic studies of related individuals. BMC Bioinformatics **2018**, 19, doi:10.1186/S12859-018-2057-X
9. Wasserman, L. *All of Statistics*; Springer Texts in Statistics; Springer New York: New York, NY, 2004; ISBN 978-1-4419-2322-6.
10. Kim, H.-Y. Statistical notes for clinical researchers: assessing normal distribution (2) using skewness and kurtosis. *Restor. Dent. Endod.* **2013**, *38*, 52–4, doi:10.5395/rde.2013.38.1.52.
11. Geraci, M.; Bottai, M. Quantile regression for longitudinal data using the asymmetric Laplace distribution. *Biostatistics* **2007**, *8*, 140–154, doi:10.1093/BIOSTATISTICS/KXJ039.
12. Harville D.; Callanan T. Computational aspects of likelihood-based inference for variance component. *Advances in Statistical Methods for Genetic Improvement of Livestock*, **1989**, 136–176 10.1007/BF01066731.
13. Vazquez A.I.; Bates D.M.; Rosa G.J.M.; Gianola D.; Weigel K.A.; Technical note: An R package for fitting generalized linear mixed models in animal breeding. *J Anim Sci.* **2010**, *88(2)*, 97-504. doi:10.2527/JAS.2009-1952
14. Bottai M.; Orsini N. A command for Laplace regression. *Stata J.* **2013**; *13(2)*, 302-314. https://doi.org/10.1177/1536867X1301300204.
15. Geraci, M. Linear Quantile Mixed Models: The lqmm Package for Laplace Quantile Regression. *J. Stat. Softw.* **2014**, *57*, 1–29, doi:10.18637/JSS.V057.I13.
16. Efron, B.; Tibshirani, R. *An Introduction to the Bootstrap*; Chapman and Hall/CRC: Boca Raton, Florida, **1994;** ISBN 9780429246593.
17. Emmert-Streib, F.; Dehmer, M. Large-Scale Simultaneous Inference with Hypothesis Testing: Multiple Testing Procedures in Practice. *Mach. Learn. Knowl. Extr. 2019, Vol. 1, Pages 653-683* **2019**, *1*, 653–683, doi:10.3390/MAKE1020039.
18. Consortium, I.M.S.G. Multiple sclerosis genomic map implicates peripheral immune cells and microglia in susceptibility. *Science (80-.).* **2019**, *365*, eaav7188, doi:10.1126/science.aav7188.
19. Seral-Cortes, M.; Sabroso-Lasa, S.; De Miguel-Etayo, P.; Gonzalez-Gross, M.; Gesteiro, E.; Molina-Hidalgo, C.; De Henauw, S.; Gottrand, F.; Mavrogianni, C.; Manios, Y.; et al. Development of a Genetic Risk Score to predict the risk of overweight and obesity in European adolescents from the HELENA study. *Sci. Rep.* **2021**, *11*, doi:10.1038/S41598-021-82712-4.
20. Iqbal, A.; Kim, Y.S.; Kang, J.M.; Lee, Y.M.; Rai, R.; Jung, J.H.; Oh, D.Y.; Nam, K.C.; Lee, H.K.; Kim, J.J. Genome-wide Association Study to Identify Quantitative Trait Loci for Meat and Carcass Quality Traits in Berkshire. *Asian-Australasian J. Anim. Sci.* **2015**, *28*, 1537–1544, doi:10.5713/AJAS.15.0752.

21. Neter, J.; Kutner, M.H.; Nachtsheim, C.J.; Wasserman, W. *Applied Linear Statistical Models, 4th Edition*; WCB McGraw Hill/Irwin: Columbus, OH, USA, **1996**; Volume 1, ISBN 0256117365.
22. Nakagawa, S.; Schielzeth, H. A general and simple method for obtaining R2 from generalized linear mixed-effects models. *Methods Ecol. Evol.* **2013**, *4*, 133–142, doi:10.1111/J.2041-210X.2012.00261.X.
